# Acrolein preferentially damages nucleolus eliciting ribosomal stress and apoptosis in human cancer cells

**DOI:** 10.18632/oncotarget.12608

**Published:** 2016-10-12

**Authors:** Hsiang-tsui Wang, Tzu-ying Chen, Ching-wen Weng, Chun-hsiang Yang, Moon-shong Tang

**Affiliations:** ^1^ Department of Pharmacology, National Yang-Ming University, Taipei, Taiwan; ^2^ Department of Environmental Medicine, Pathology and Medicine, New York University School of Medicine, Tuxedo Park, NY, USA

**Keywords:** acrolein, DNA damages, rDNA/ rRNA, ribosomal stress/ nucleolar stress, RPL11-MDM2-p53

## Abstract

Acrolein (Acr) is a potent cytotoxic and DNA damaging agent which is ubiquitous in the environment and abundant in tobacco smoke. Acr is also an active cytotoxic metabolite of the anti-cancer drugs cyclophosphamide and ifosfamide. The mechanisms via which Acr exerts its anti-cancer activity and cytotoxicity are not clear. In this study, we found that Acr induces cytotoxicity and cell death in human cancer cells with different activities of p53. Acr preferentially binds nucleolar ribosomal DNA (rDNA) to form Acr-deoxyguanosine adducts, and induces oxidative damage to both rDNA and ribosomal RNA (rRNA). Acr triggers ribosomal stress responses, inhibits rRNA synthesis, reduces RNA polymerase I binding to the promoter of rRNA gene, disrupts nucleolar integrity, and impairs ribosome biogenesis and polysome formation. Acr causes an increase in MDM2 levels and phosphorylation of MDM2 in A549 and HeLa cells which are p53 active and p53 inactive, respectively. It enhances the binding of ribosomal protein RPL11 to MDM2 and reduces the binding of p53 and E2F-1 to MDM2 resulting in stabilization/activation of p53 in A549 cells and degradation of E2F-1 in A549 and HeLa cells. We propose that Acr induces ribosomal stress which leads to activation of MDM2 and RPL11-MDM2 binding, consequently, activates p53 and enhances E2F-1 degradation, and that taken together these two processes induce apoptosis and cell death.

## INTRODUCTION

Acrolein (Acr) is a ubiquitous environmental contaminant that predominantly arises from incomplete combustion such as cooking and tobacco smoking [1]. Acr contains a carbonyl group and an α,β-unsaturated double bond which owing to their reactivity with different cellular components such as nucleic acids and proteins can induce mutagenic DNA adducts and induce protein dysfunction [2]. Acr has been proposed to be carcinogenic via DNA adduct induction and impairment of DNA repair function [2–5]. In addition, Acr also has a potent cytotoxic effect; it can induce cell death via both apoptosis and necrosis pathways [1, 6, 7]. Acr has been shown to be a major cause of tobacco smoke related chronic obstructive pulmonary diseases (COPD) and asthma [8]; it has been proposed that the apoptotic and necrotic effects of Acr elicits these diseases [6, 7].

Acr is a major metabolite of the antitumor drugs cyclophosphamide and ifosfamide. Acr cytotoxicity is believed to be the major antitumor activity of these drugs [9, 10]. Hence, understanding the Acr-induced effects - DNA adduct formation, protein modifications, and cell death - may enhance not only our understanding of how Acr induces different diseases but also help to elucidate the anti-tumor activity of these drugs. While it is well understood of how Acr adducts DNA and proteins, the cellular processes by which Acr elicits cell death are not clear.

Mapping Acr-induced DNA adduct formation at the DNA sequence level we have found that Acr-DNA adducts are preferentially formed at GC rich sequences [2–5]. This finding raises the possibility that the nucleolus is also a preferential target of Acr since ribosomal DNA (rDNA) in nucleolus is GC rich [11]. If this is the case, then it is possible that Acr-rDNA binding elicit cell death signals since it is well established that rDNA damage is the major cellular stress response hub [12, 13]. In this study we tested this possibility and delineate the Acr-induced stress pathway. Using an immunofluorescent staining method, we found that Acr-DNA adducts are indeed preferentially formed in the nucleolus. Acr induces oxidative damage in both rDNA and rRNA. Acr interrupts rRNA transcription and processing, as well as polysome formation and global protein translation.

It is well understood that the nucleolus is the site of ribosome biogenesis which is an essential and energy consuming cellular process [12, 13], and that impairment of ribosome biogenesis causes ribosomal stress (also known as nucleolar stress) [12, 14, 15]. The correlation of the DNA damage response with the nucleolus has shown that the nucleolus acts as a sensor for cellular stress signals through stabilization of p53 by ribosomal protein (RP)–MDM2/HDM2 interactions, which induces cell cycle arrest or apoptosis [14, 16–20]. Intriguingly, we found that Acr induces the same extent of apoptosis and cell death in human lung adenocarcinoma A549 cells and human cervical cancer HeLa cells with active p53 and inactive p53, respectively. These results raise the question of “what are the apoptosis signals induced by Acr in these cells?”.

We found that Acr induces ribosomal stress resulting in disintegration of ribosome, and enhancing RP11-MDM2 interactions. Consequently, Acr reduces binding of activated p53 proteins in A549 cells, and reduces binding of E2F-1 with MDM2 causing E2F-1 degradation in A549 and HeLa cells. We propose that Acr cytotoxicity occurs via ribosomal stress which activates p53 and enhances E2F-1 degradation, both of which can cause cell death.

## RESULTS

### Acrolein (Acr) induces cytotoxicity and apoptosis in human cancer cells

**Figure 1:**
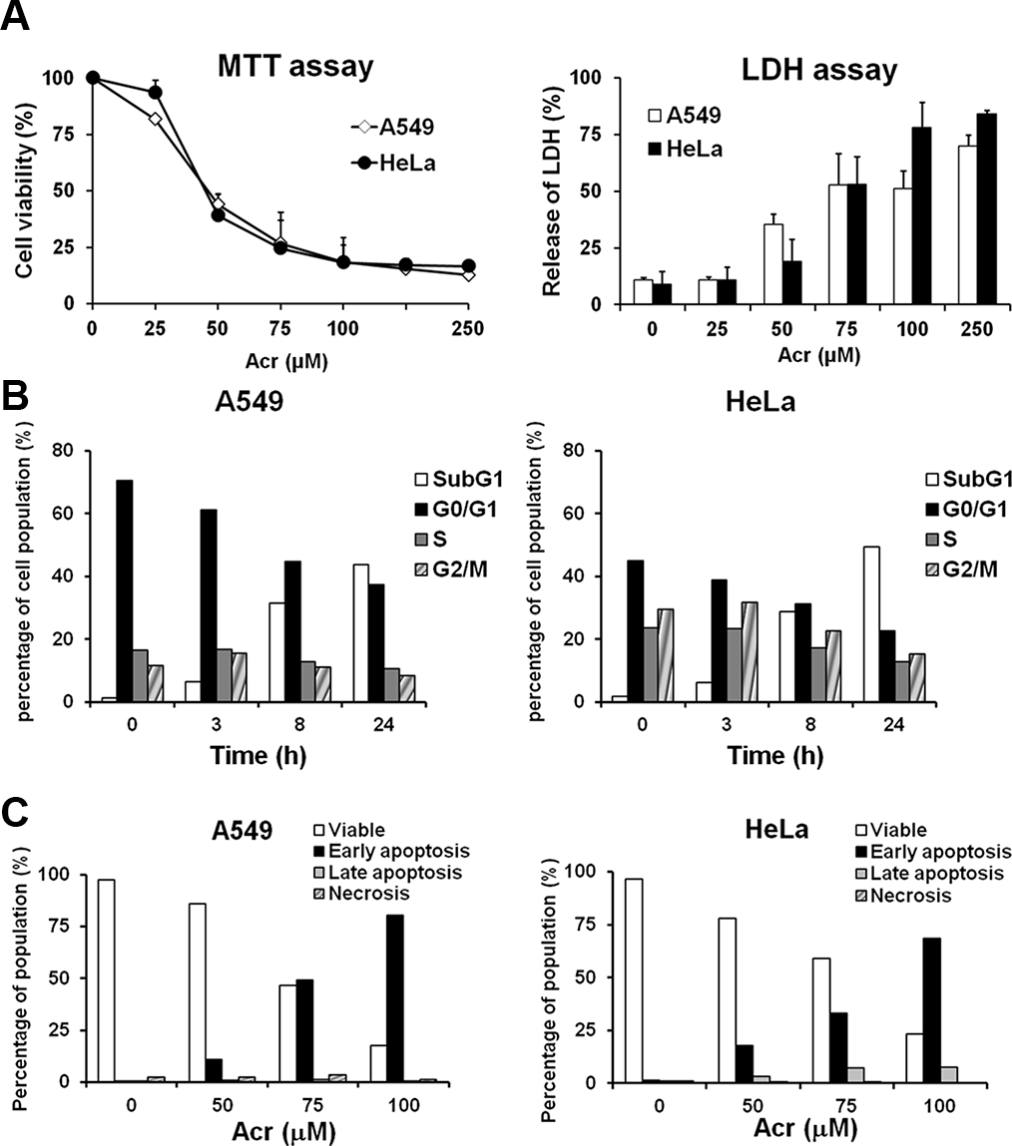
Acrolein induces the same cytotoxic effect in A549 and HeLa cells (**A**) Exponentially growing A549 and HeLa cells were treated with different concentrations of Acr (0–250 μM) for 24 h and the cell survival was determined by MTT assay (left panel) and LDH assay (right panel) as described in Materials and Methods. (**B**) Flow cytometric DNA profiles after propidium iodide (PI) staining of HeLa and A549 cells treated with Acr (75 μM, 0–24 h). The percentage of cell population in each cell cycle phase represents the mean of three different experiments. (**C**) Flow cytometry analysis of apoptosis/ necrosis by Annexin V and PI staining in Acr-treated A549 and HeLa cells (0–100 μM, 24 h).

Although Acr induces cell death via both apoptosis and necrosis process, the signals that induce these processes are not well understood. It has been established that Acr mediates antitumor activity of cyclophosphamide and ifosfamide through its cytotoxicity [9, 10]. Since p53 plays a center role in apoptosis and 50% of human cancer cells either have inactive p53 function or carry mutant p53, it is important to determine the toxicity of Acr in cells with different activities of p53 and the cell death mechanisms. We chose to determine the Acr induced cytotoxicity and cell death processes in human lung adenocarcinoma cells A549 which carry wild type p53 gene and human cervical cancer HeLa cells in which p53 is inactive [21, 22]. The result in Figure 1A shows that both cells have the same sensitivity toward Acr-induced cytotoxicity. We also found that Acr induces mainly apoptosis in A549 and HeLa cells (Figure 1B and 1C). These results indicate that Acr induces both p53 dependent and independent apoptosis.

### Acrolein induces Acr-dG adducts and 8-oxo-dG adducts preferentially in nucleoli

**Figure 2:**
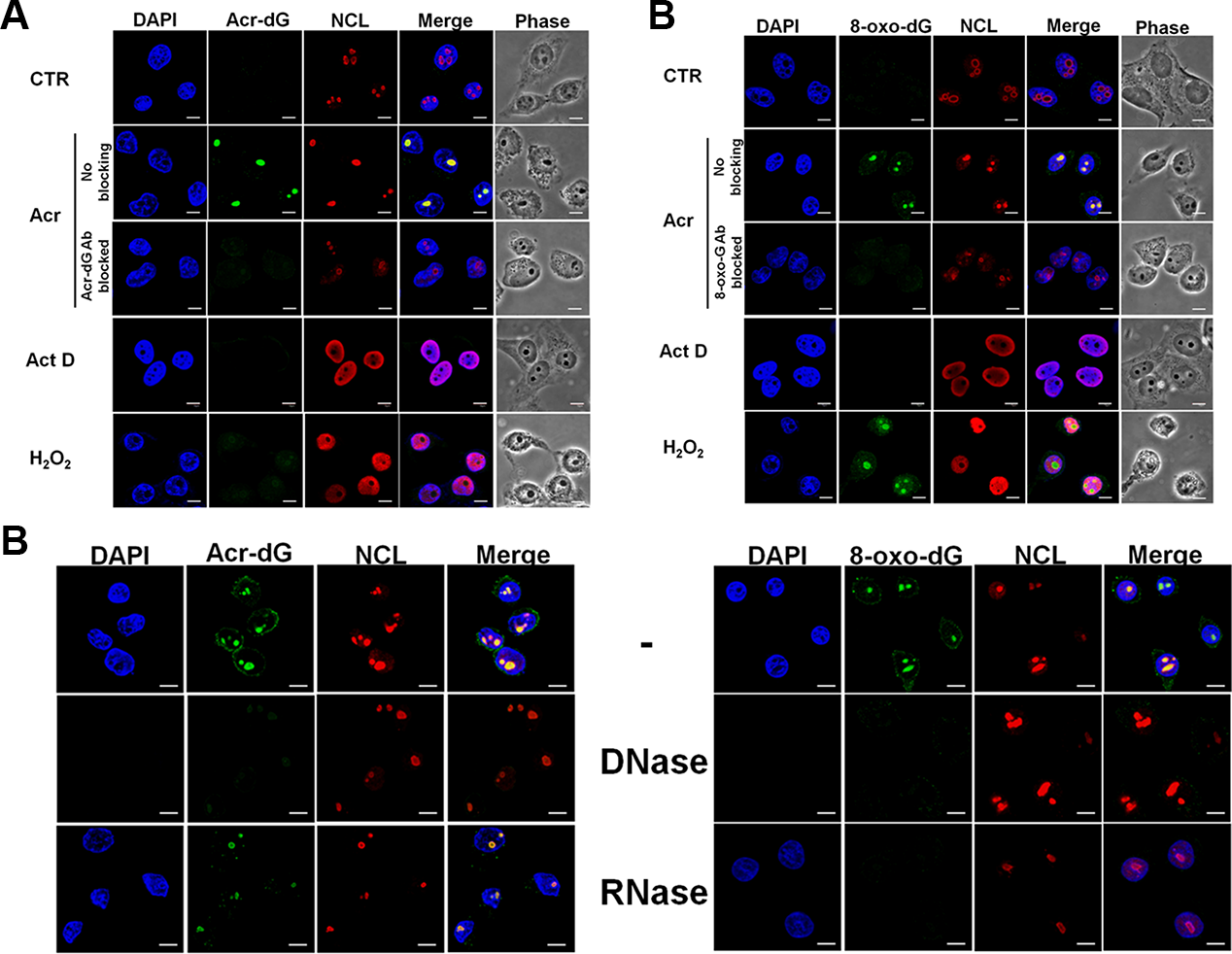
Acrolein induces Acr-dG adducts and 8-oxo-dG adducts in nucleoli HeLa cells were treated with Acr (75 μM, 3 h), Act D (20 ng/ml, 3 h) or H_2_O_2_ (1 mM, 3 h), fixed, stained with (**A**) anti-Acr-dG and (**B**) anti-8-oxo-dG antibody followed by goat anti-mouse FITC-conjugated secondary antibody and then examined by microscopy. Nucleolin (NCL) was used to stain nucleoli (A and B). The specificity of Acr-dG or 8-oxo-dG antibody was also confirmed by pre-incubating these antibodies with a 15 to 20-fold excess of soluble Acr-dG and 8-oxo-dG adducts. (**C**) DNase and RNase treatment in Acr-treated HeLa cells was described in Materials and Methods. Scale bar: 10 mm. Note: RNAse digests RNA containing oxidative DNA damage recognized by 8-oxo-dG antibody but not DNA (DAPl stained) indicating that Acr-induces 8-oxo-G adducts in RNA.

Previously, we found that Acr can damage genomic DNA to induce mutagenic Acr-dG adducts in different human cells, and that Acr-dG adducts preferentially occurred in run's of G sequences [4, 5, 23]. Since rDNA is rich in GC content it is possible that rDNA in the nucleolus is a preferential target for Acr [15, 24–26]. Using an immunofluorescence assay with a specific anti-Acr-dG antibody, we found that Acr-dG adducts were preferentially observed in the nucleoli in both A549 and HeLa cells (Figure 2A and Figure S1A). It has been found in cell culture that, Acr can trigger lipid peroxidation and production of intracellular reactive oxygen species, as shown in Figure S2, which can induce oxidative DNA damage [27]. Results in Figure 2B show that Acr indeed induces DNA damage that was recognized by anti-8-oxo-dG antibodies (Figure S1B). The formation of Acr-dG and 8-oxo-dG in nucleoli was further confirmed by the results in Figure 2A and 2B and Figure S1A and S1B showing that pre-incubating anti-Acr-dG or anti-8-oxo-dG antibody with a 15 to 20-fold excess of soluble Acr-dG and 8-oxo-dG abolished their ability to detect the Acr-dG or 8-oxo-dG formation in the nucleoli.

Since the nucleolus is a large aggregate consisting of rDNA, precursor and mature rRNAs, it is possible that in addition to rDNA, the rRNA is also a target of Acr [12]. To test this possibility, using DNase and RNase treatment followed by the immunofluorescence assay with anti-Acr-dG or anti-8-oxo-dG, we found that the 8-oxo-dG antibody recognizing oxidative damage was sensitive to both DNAse and RNAse digestion, the same as damage induced by H_2_O_2_. These results indicate that Acr-induced oxidative damage can occur in both rDNA and rRNA (Figure 2C and Figure S1C).

**Figure 3:**
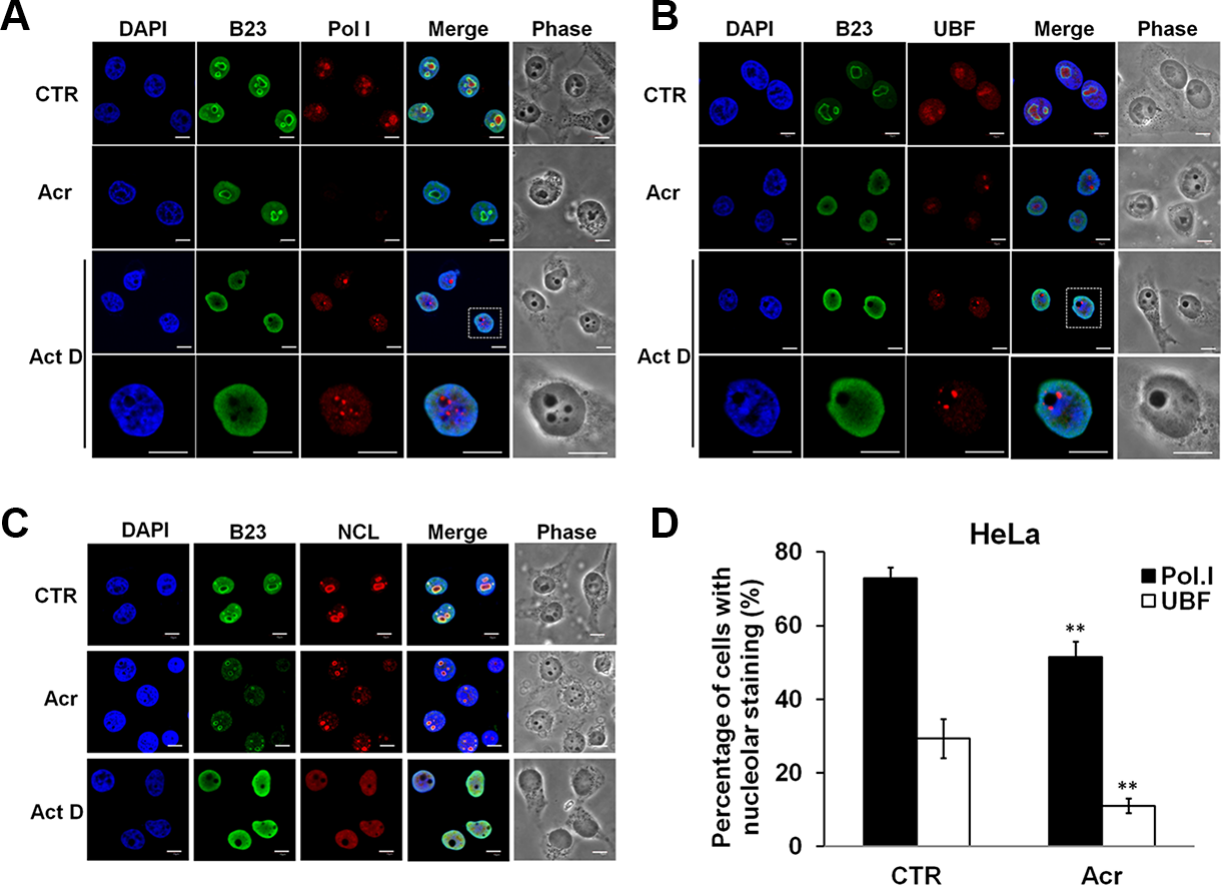
Acrolein decreases nucleolar RNA polymerase I (Pol I) and UBF translocation and induces nucleolar disintegration (**A** and **B**) Immunofluorescence staining of RNA Pol I and UBF in Acr- and Act D-treated HeLa cells. Cells were treated with Acr (75 μM, 3 h) or Act. D (20 ng/ml, 3 h), fixed, stained with RNA Pol I and UBF antibody followed by goat anti-rabbit Rhodamine-conjugated secondary antibody and then examined by microscopy. B23 was used to stain nucleoli. The magnification of Act D-treated cells in white dashed rectangular box was shown in the lowest row. Scale bar: 40 mm. (**C**) Visualization of nucleolar structure in HeLa cells treated with Acr (75 μM) or Act D (20 ng/ml) for 3 h using immunofluorescence staining of B23 and NCL antibody. Nuclei were counter-stained with DAPI. Scale bar: 10 mm. Quantifications of nucleolar Pol I and UBF are showed in (**D**). Histograms show the values (mean ± s.d.) of three independent experiments. *P value < 0.05, **P value < 0.01. Student's *t*-test was used to calculate significance between control and treatment.

We also determined the effect of Acr treatment on nucleolus morphology and molecular re-arrangements and compared these changes to those induced by actinomycin D (Act D), a well-characterized nucleolar disruptor [14]. Results in Figure 3 show that Act D treatment (20 ng/ml, 3 h) induced RNA polymerase I (Pol I) or upstream binding factor (UBF) segregated into caps around the DAPI-sparse nucleoli such that nucleophosmin (B23) and nucleolin (NCL) were mislocalized over the nucleoplasm (Figure 3A and 3B and Figure S3A and S3B). Acr (75 μM 3 h) induced movement of RNA Pol I or UBF, but not onto the nucleolar cap (Figure 3D and Figure S3D). Rather, Acr induced translocation of B23 into the nucleoplasm was only observed in cells treated with Acr for a short period (3 h) (Figure 3C and Figure S3C). These results raise the possibility that Acr may have effects on different levels of rRNA synthesis, including inhibition of rRNA processing.

### Acrolein interrupts the synthesis of rRNA, ribosome assembly and global translation but not rRNA processing

**Figure 4:**
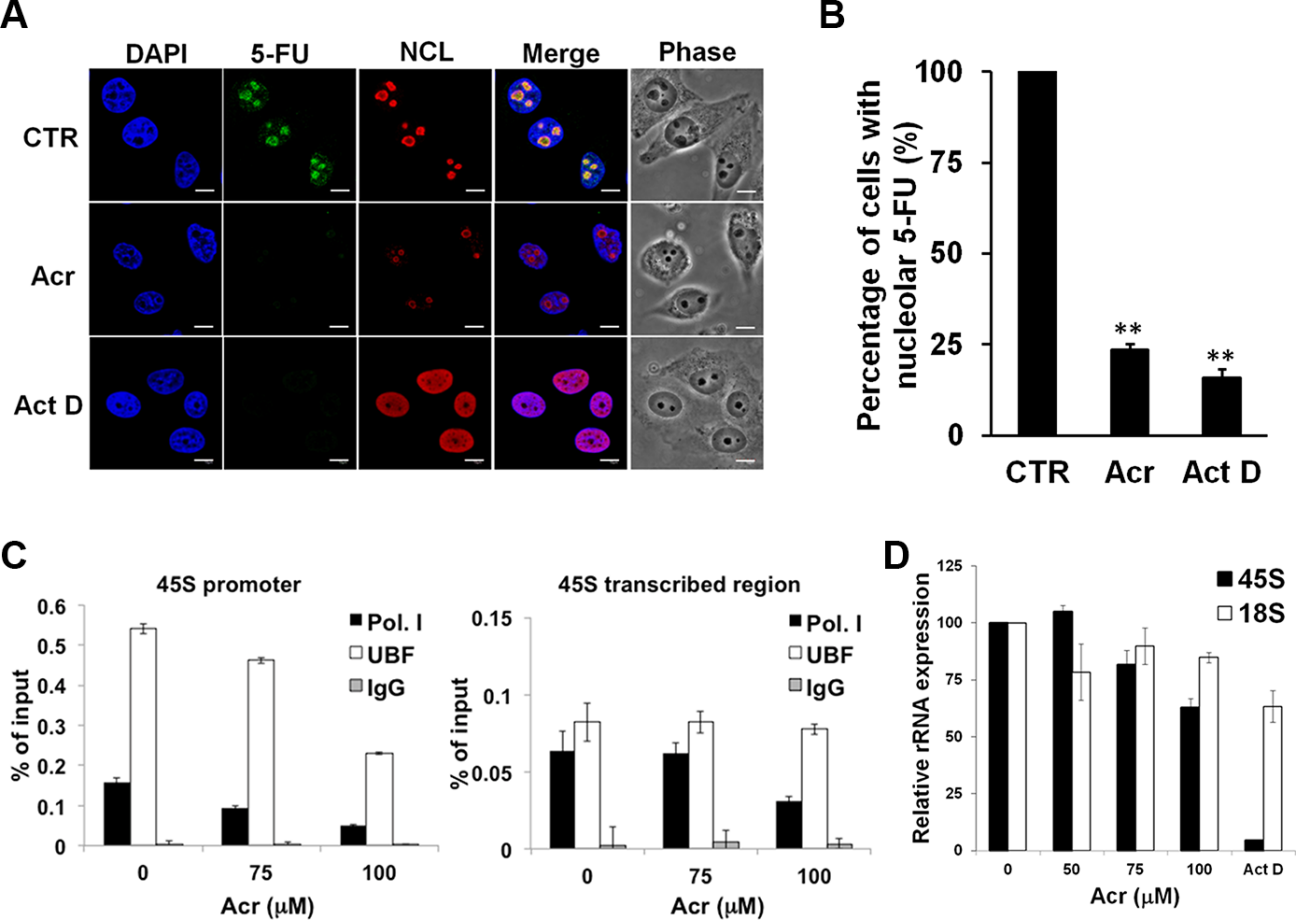
Acrolein interrupts the synthesis of rRNA through inhibition of transcriptional activation of RNA Pol I and UBF (**A**) Acr inhibits RNA synthesis in nucleoli. HeLa cells were treated with Acr (75 μM) or Act D (20 ng/ml) for 3 h, labeled newly synthesized RNA with 5-fluorouridine (5-FU) for 15 min, and 5-FU labeld RNA's were labeled with specific FITC-conjugated monoclonal antibodies. Nucleolin (NCL) was used to stain nucleoli and DAPI counter-stained nuclei. Scale bar: 10 mm. Quantifications of nucleolar 5-FU staining is shown in (**B**). Histograms show the values (mean ± s.d.) of three independent experiments. *P value < 0.05, **P value < 0.01. Student's *t*-test was used to calculate significance between control and treatment. (**C**) ChIP assay for RNA Pol I and UBF binding at the promoter region and the transcribed region of 45S rRNA in HeLa cells treated with Acr (75 μM, 3 h). (**D**) Evaluation of 45S and 18S rRNA expression in HeLa cells treated with Acr (0–100 μM, 3 h) using real-time RT-PCR analysis.

Previous studies have shown that inhibition of rRNA synthesis is related to disintegration of nucleolar structures [28]. Using immunofluorescence detection we found that Acr treatment diminished nucleolar 5-fluorouridine incorporation into nascent rRNA indicating that Acr treatment inhibits rRNA synthesis (Figure 4A and 4B and Figure S4A). Chromatin immunoprecipitation assay results in Figure 4C show that Acr treatment decreased binding of RNA Pol I and UBF on the promoter region of rDNA in cells treated with Acr. These results are consistent with the decreased co-localization of RNA Pol I and UBF showing in Figure 3D and Figure S3D. The quantitative real-time RT-PCR analysis results show that Acr decreased the expression of 45S pre-rRNA, but had no effect on 18S rRNA expression (Figure 4D and Figure S4C). This result indicated that while Acr inhibited the synthesis of rRNA, it impaired the processing of rRNA only modestly.

**Figure 5:**
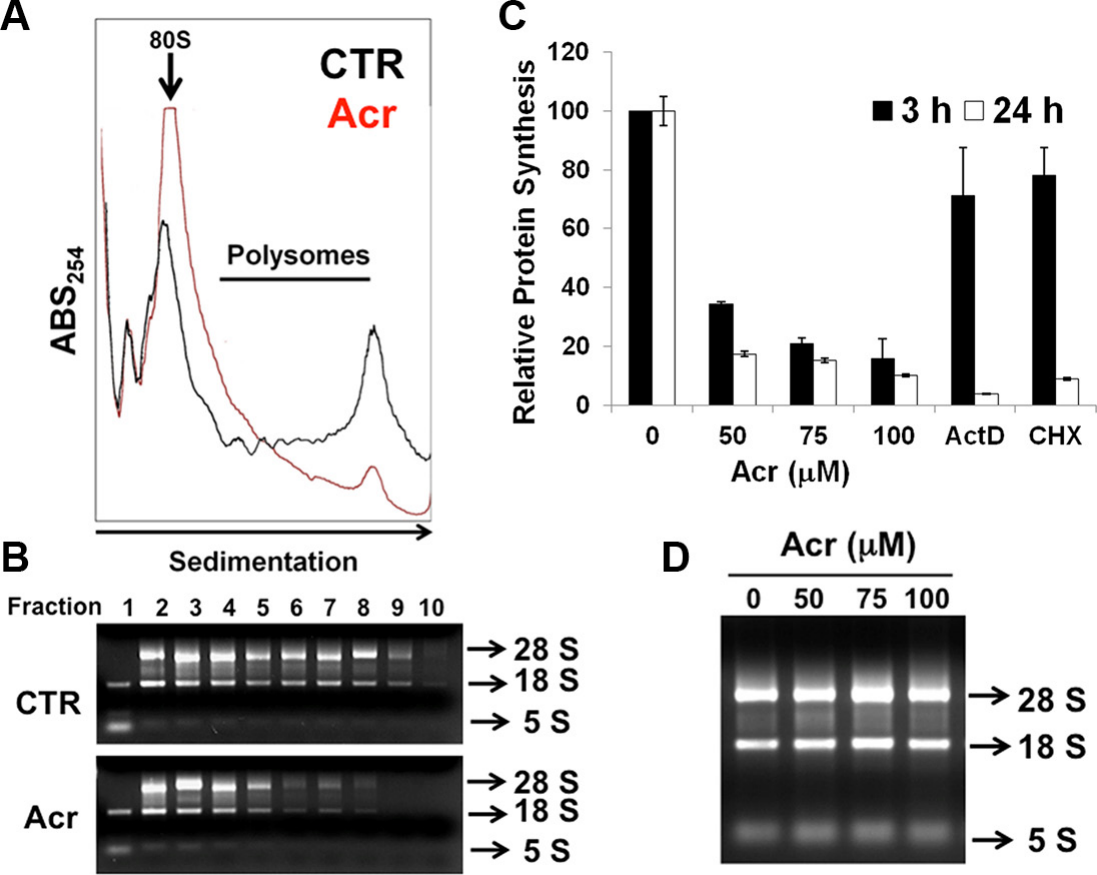
Acrolein interferes with ribosomal assembly and global protein synthesis (**A**) Effect of Acr treatment (75 μM, 3 h) on the distribution of monosomes and polysomes in HeLa cells. The monsomes (80S) and polysomes (indicated by a bar) were separated by sucrose gradient centrifugation and each fraction was measured by absorption at 254 nm. (**B**) RNA extracts from different fractions were analyzed by gel electrophoresis. (**C**) Effect of Acr (0–100 μM), Act D (20 ng/ml) or cyclohexymide (CHX) (50 μM) for 3 or 24 h treatment on total protein synthesis. HeLa cells were treated with Acr (0–100 μM), Act D (20 ng/ml) or cyclohexymide (CHX) (50 μM) for 3 or 24 h, and then with puromycin analog o-propargyl-puromycin (OPP) for 30 min. OPP incorporation at the C-terminus of translating polypeptide chains, stops translation. These truncated C-terminal alkyne -labeled proteins were then subsequently detected *via* copper-catalyzed click chemistry using 5 FAM-Azide followed by flow cytometry. Histograms show the values (mean ± s.d.) of three independent experiments. (**D**) Gel electrophoresis of total rRNA in HeLa cells treated with Acr (0–100 μM, 3 h).

In order to further understand whether Acr influenced ribosome biogenesis, we used the ribosomal profiling assay to assess whether the large and small ribosomal subunits could be assembled normally to perform their respective translation functions. These results in Figure 5A and 5B and Figure S4B show that indeed Acr dramatically diminished the formation of polysomes (Figure 5A and 5B and Figure S4B). Consistent with this reduction of polysome formation, Acr treatment also caused a dose-dependent decrease of global protein synthesis in both A549 and HeLa cells. (Figure 5C and Figure S4D). However, no difference in the amount of 28S and 18S rRNAs was found among cells treated with or without Acr (Figure 5D). Together, these results suggest that Acr inhibited global mRNA translation by interfering with rRNA synthesis (reduction of Pol I and UBF loading at 45S promoters) and consequentially polysome assembly in both A549 and HeLa cells.

### Acrolein stabilizes/ activates p53 in p53-active A549 cells

**Figure 6:**
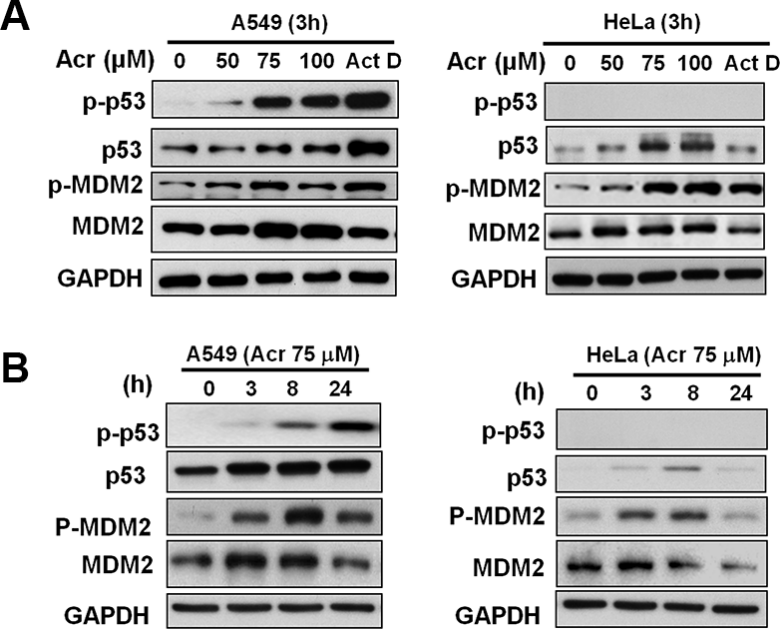
Acrolein stabilizes and activates p53 in p53-active A549 cells (**A**) Representative western blot of total MDM2, p53, and the phosphorylated form of MDM2 (p-MDM2, Ser166) and p53 (p-p53, Ser15) expression in control and Acr (0–100 μM, 3 h)-treated A549 and HeLa cells. (**B**) Time course of total MDM2, p53, p-MDM2, and p-p53 expression in A549 and HeLa cells treated with Acr (75 μM, 0–24 h). Note: Acr treatment increases p-53 in a concentration and time dependent fashion In A549 cells but not in HeLa cells.

It is well established that nucleolar transcription is inhibited under DNA damage induced stress [12, 14, 15], during which several proteins regulate rRNA transcription or processing. The nucleolus acts as a sensor for cellular stress signals through stabilization of p53 by RP–Mdm2/HDM2 and ARF–Mdm2/HDM2 interactions, which induce cell cycle arrest or apoptosis [16–19]. We found that Acr treatment caused an increase of both phosphorylated and total p53 protein levels in p53-active A549 cell in dose and time-dependent manner (Figure 6). The total protein and the phosphorylated MDM2 levels were also increased up to 8 h incubation. After 24 h incubation the levels of MDM2 and phosphorylated MDM2 decreased while the levels of p53 and phosphorylated p53 continuously increased. These results indicate that the decrease of MDM2 is due to a p53-MDM2 feedback loop and that p53 is a sensor for Acr-induced ribosomal stress via MDM2 activation.

### Acrolein enhances expression of MDM2 and phosphorylation of MDM2 in HeLa cells with inactive p53

Since Acr also induces ribosomal stress in HeLa cells which have p53 nullified by viral E6 [21, 22], we then determined the signal pathway of this ribosomal stress in p53-inactive cells. Results in Figure 6 show that while Acr treatment modestly increase total p53 levels after no phosphorylated p53 was detected. Acr also failed to stimulate the expression of its downstream targets, p21 in these p53 inactive cells (data not shown). These results indicate that Acr-induced ribosomal stress does not activate p53. However, Acr treatment enhances both total and phosphorylated MDM2 indicating that MDM2 play a p53 independent role in Acr-induced ribosomal stress response.

### Acrolein induces E2F-1 degradation in p53-active A549 and p53-inactive HeLa cells

**Figure 7:**
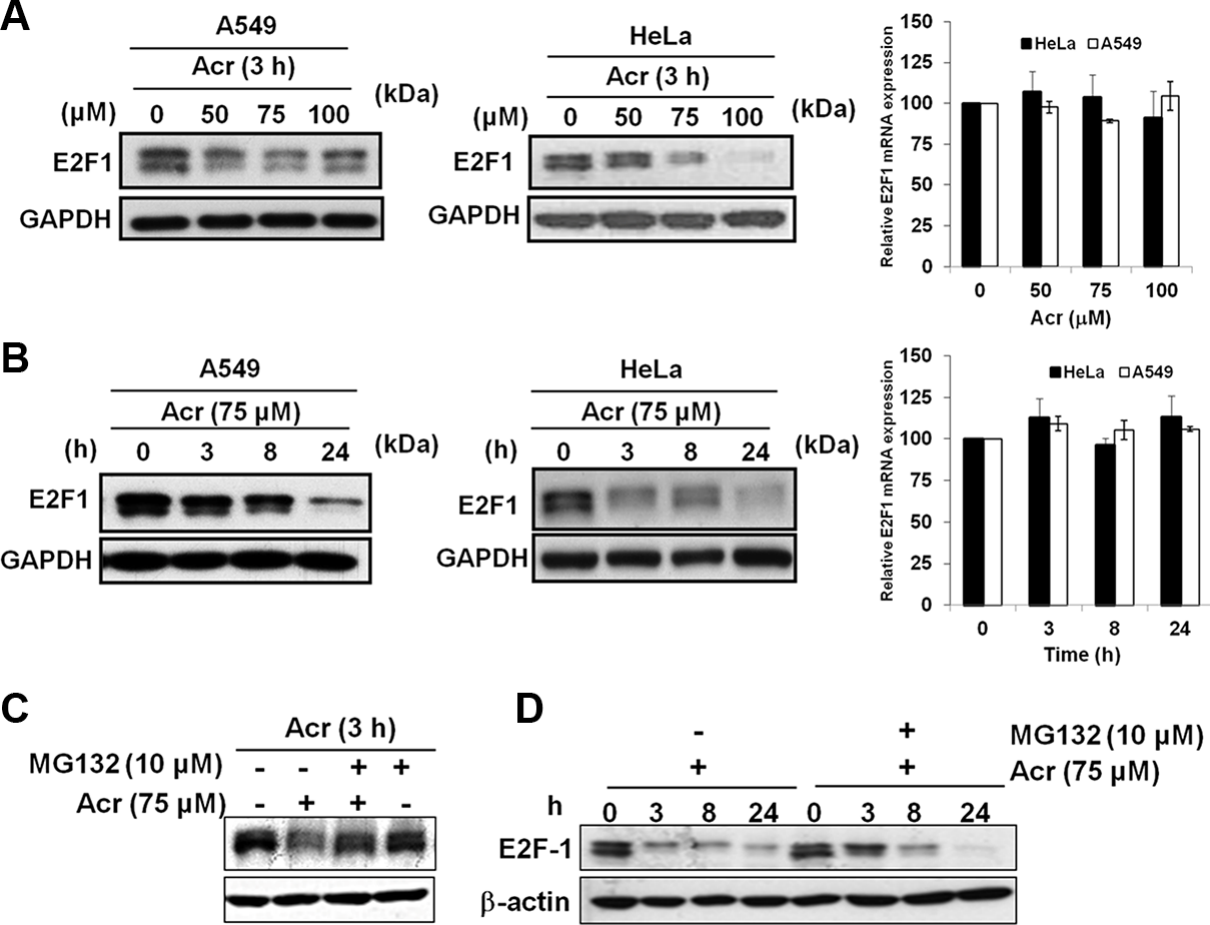
Acrolein increases proteasomal degradation of E2F-1 in p53-active A549 and p53-inactive HeLa cells (**A**) Effect of Acr treatment (0–100 μM, 3 h) on E2F-1 expression in A549 and HeLa cells. Protein levels detected by Western blots are shown in the left and mRNA levels detected by real-time RT-PCR are shown at right. Values are mean ± s.d. of three experiments. (**B**) Time course of E2F-1 protein and mRNA expression in A549 and HeLa cells treated with Acr (75 μM, 0–24 h). Representative Western blot E2F-1 protein is shown on the left and real-time RT-PCR quantification of E2F-1 mRNA is shown on the right. Values are mean ± s.d. of three experiments. (**C**) Western blot showing E2F-1 protein expression in control and acrolein-treated HeLa cells (0–100 μM, 3 h) either pre-treated or not treated with MG-132 (10 μM, 2 h). (**D**) Time course analysis of E2F-1 protein expression in control and Acr-treated HeLa cells (75 μM, 0–24 h) either pre-treated or not treated with MG-132 (10 μM, 2 h).

Since E2F-1 is also known to be involved in regulating rRNA transcription and coordinating DNA damage and nucleolar stress [29], we next measured the expression levels of E2F-1 in Acr-treated A549 and HeLa cells. As can be seen (Figure 7A and 7B), E2F-1 was reduced in a time-dependent fashion in A549 and HeLa cells. However, no change in the mRNA levels of E2F-1 occurred at that time (Bar graphs of Figure 7A and 7B), indicating a post-transcriptional mechanism for the downregulation of the E2F-1 protein. The reduction of E2F-1 protein in Acr-treated cells was partially restored by MG-132, a well-known proteasome inhibitor (Figure 7C and 7D). These results suggest that Acr directly and/or indirectly via ribosomal stress response caused E2F-1 protein degradation via a proteasome pathway.

### Acrolein increases the binding of ribosomal protein L11 (RPL11) with MDM2, resulting in stabilization of p53 and degradation of E2F-1

**Figure 8:**
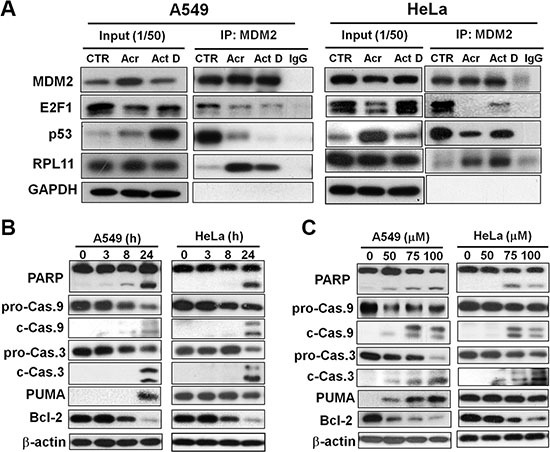
Acrolein induces binding of RPL11 to MDM2, p53 stabilization, degradation of E2F-1 and Bcl2, and activates apoptotic enzymes caspase 9 and 3 (**A**) Effects of Acr treatment (75 μM, 6 h) on the binding of MDM2 with RPL11 p53 and E2F-1 were determined by using an immunoprecipitation method. Cell lysates of Acr-treated A549 and HeLa cells were immunoprecipitated with anti-MDM2 polyclonal antibodies (MDM2-IP), followed by immunoblotting with anti-MDM2, p53, E2F-1, and RPL11 antibodies. For each lysate, 20% of the quantity used for immunoprecipitation was loaded as input control (input). (**B** and **C**) Dose effect and time course of Acr effects on cleavage of PARP, and procaspase 9 and 3 (pro-Cas 9 to c-Cas 9; pro-Cas 3 to c-Cas 3), PUMA induction and degradation of Bcl-2 in A549 and HeLa cells. For time course analysis cells were treated with Acr (75 μM) and incubated for different time periods (C) and for dose effect cells were treated with different concentrations of Acr and incubated for 24 h (B). Symbols: p-Cas 9, procaspase 9, c-Cas 9, cleaved caspase 9; p-Cas 3, procaspase 3, c-Cas 3, cleaved caspase 3.

The mechanism of p53 stabilization (Figure 6) or E2F-1 degradation (Figure 7) after perturbation of ribosome biogenesis is possibly the consequence of changes in functional and physical interactions of these proteins with MDM2. Previous studies have shown that MDM2 negatively controls p53 activity in two ways: by binding to the protein and interfering with its transactivation activity, and by facilitating p53 proteasomal degradation thereby acting as an E3 ubiquitin ligase [30]. On the other hand, it has been shown that MDM2 binds to the E2F-1 protein and protects it from proteasome-mediated degradation [31]. As a consequence of reduced ribosome biogenesis, several RPs such us L5, L11, L23 and S7 are no longer used for ribosome generation but instead binding to MDM2 to relieve its inhibition on p53, as well as its protection toward E2F-1 [32–36]. Interestingly, our results showed that in Acr-treated A549 cells, the amounts of p53 and E2F-1 complexed with MDM2 were markedly reduced in comparison with controls cells (Figure 8A). By contrast, the amount of RPL11 associated with MDM2 was significantly increased in Acr-treated cells (Figure 8A). We interpret these results as indicating that Acr-induced rRNA synthesis inhibition can cause a disintegration of the polysome and the integrity of ribosomal structure. Consequently, ribosomal proteins including RPL11 will be released from the ribosomal structure. RPL11 is able to bind to MDM2. The RPL11 bound MDM2 loses its function in mediating p53 degradation and protecting E2F-1 against proteasomal degradation.

Considering that both p53 and E2F-1 are crucial regulators of cell apoptosis, we next examined the subsequent signaling responsible for Acr-induced apoptosis in both A549 and HeLa cells observed in Figure 1B and 1C. As can be seen, a time and dose-dependent cleavage of caspase 3, caspase 9, and PARP were observed in these cells (Figure 8B and 8C), suggesting apoptosis induced by Acr. Interestingly, PUMA, a pro-apoptotic gene induced by p53 [37] was increased in dose and time-dependent manner in p53-active A549 cells, but not in p53-inactive HeLa cells. However, Bcl-2, an anti-apoptotic gene regulated by E2F1 [38] was decreased in dose and time-dependent manner in both cells. This indicates that Bcl-2 plays a major role in Acr-induced apoptosis in p53-inactive HeLa cells.

### Acr induces similar effects on apoptosis, MDM2 phosphorylation and E2F-1 reduction in p53-knockdown A549 cells as in HeLa cells

**Figure 9:**
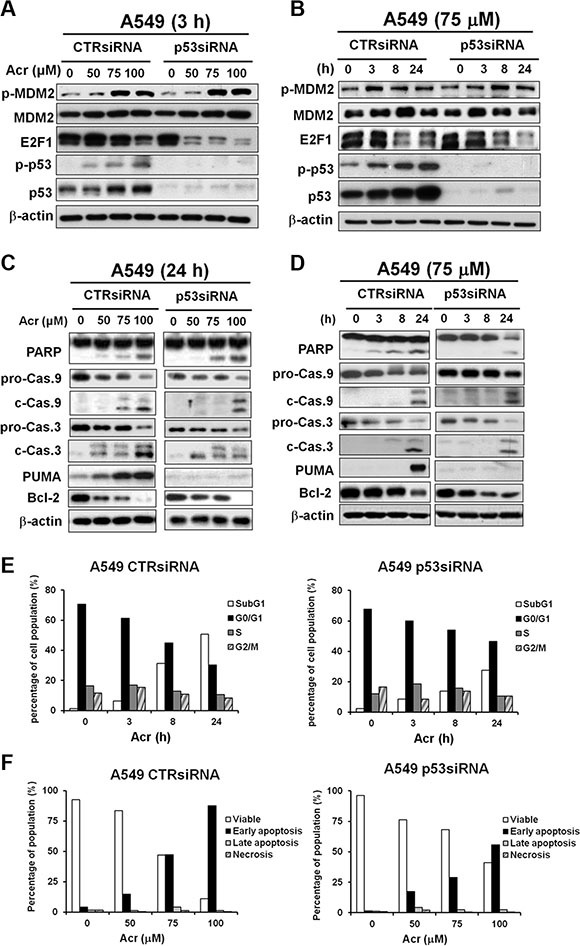
Acrolein induces apoptosis in A549 cells with knockdown of p53 via E2F1 degradation A549 cells transfected with control siRNA and p53 siRNA were treated with different concentrations of Acr (0–100 μM, 3h), or treated with Acr (75 μM) for different time periods. Expression of (**A** and **B**) MDM2, p53, E2F-1, (**C** and **D**) PARP, caspase 9, caspase 3 (pro-Cas 9 to c-Cas 9; pro-Cas 3 to c-Cas 3), PUMA and Bcl-2 were detected in these cells as described in Materials and Methods. (**E**) Flow cytometric DNA profiles after propidium iodide (PI) staining of A549 cells with control siRNA and p53 siRNA treated with Acr (75 μM) for different time periods. The percentage of cell population in each cell cycle phase represents the mean of three different experiments. (**F**) Flow cytometry analysis of apoptosis/ necrosis by Annexin V and PI staining in Acr-treated A549 cells with control siRNA and p53 siRNA (0–100 μM, 24 h). Symbols: p-Cas 9, procaspase 9, c-Cas 9, cleaved caspase 9; p-Cas 3, procaspase 3, c-Cas 3, cleaved caspase 3.

Since A549 and HeLa cells are derived from lung and cervical cancer, respectively, it is possible that apoptosis pathway induced by Acr is due to cell differences, not p53 activity. In order to further confirm the role of p53 in Acr-induced apoptosis pathways, we used siRNA to knockdown p53 in A549 cells. Results in Figure 9A and 9B show that Acr induced phosphorylation of MDM2 and reduction of E2F1 in p53-knockdown cells, which is similar to HeLa cells (Figure 6). Results in Figure 9C and 9D show that Acr can also induce apoptosis pathway in p53 knockdown cells, but the extent of apoptosis is much lower than in A549 shown in Figure 9E and 9F. Furthermore, Acr causes a decrease of Bcl-2 in these cells (Figure 9C and 9D). Taken together, these results indicate that via Bcl-2 regulation p53 plays a crucial role in Acr-induced apoptosis in p53-active cells.

## DISCUSSION

Although Acr is a ubiquitous environmental contaminant, it also is an active cytotoxic metabolite of the anti-cancer drugs cyclophosphamide and ifosfamide. It is generally accepted that the antitumor activity of these drugs resides in the cytotoxicity of their major metabolite Acr [9, 10]. However, the mechanisms via which Acr exerts its anti-cancer activity and cytotoxicity are not clear. Our previous studies have shown that Acr-dG adducts were preferentially formed in DNA runs of G sequences [4, 5, 23]. The rDNA is organized in the form of tandem repeats with high GC content in the nucleolus and we demonstrate here that indeed the nucleolus is a preferential target of Acr. It is well established that the nucleolus is the major hub for sensing DNA damage-induced stress [12, 13]. In this study we found that Acr-induced ribosomal stress cascades via disintegration of the polysome and ribosomal structure, freeing the ribosomal proteins (Figures 3 and 8). Consequently, the freed ribosomal protein RPL11 binds to MDM2 to cause MDM2 dysfunction. In p53-active A549 cells, the activated p53 proteins are stabilized which cause E2F-1 degradation. In p53-inactive HeLa cells, MDM2 proteins are unable to prevent E2F-1 from proteosomal degradation due to RPL11 binding. Reduction of E2F-1 prevents activation of cell cycling and sensitizes cells to DNA damage-induced cell death (Figure 7). We believe these two processes are the major causes of Acr cytotoxicity in human cancer cells with different activities of p53 (Figure 9).

We found that Acr induces not only Acr-dG adducts but also 8-oxo-dG adducts in rDNA. These results are consistent with previous studies showing that Acr can trigger lipid peroxidation to induce reactive oxygen species which can cause additional oxidative DNA damage [27]. This conclusion was further supported by the result that DNase treatment eliminates all sites recognized by the 8-oxo-dG antibody in the nucleolus. It has been found that oxidative damage can also occur in RNA [39–41]. Results from RNase treatment in Acr-treated cells show that while RNase treatment does not affect the integrity of nuclear DNA it eliminates all 8-oxo-dG antibody recognition sites in the nucleolus. We believe that this is due to the conformation of 8-oxo-dG containing DNA in the “A” form similar to RNA conformation [42], therefore being sensitive to RNase digestion. Our results are consistent with previous findings that RNA is vulnerable to oxidative damage [39–41]. Oxidative modification of RNA results in disturbance of the translational process and impairment of protein synthesis, which can cause cell deterioration or even cell death [43, 44]. Maintaining the integrity of rDNA is crucial for rRNA synthesis which is the initial step of ribosome biogenesis.

Previous studies have shown that inhibition of rRNA transcription and early rRNA processing steps, but not of late rRNA processing steps, coincides with the loss of nucleolar integrity [28]. Translocation of B23 was only observed at the short exposure time (3 h) for Acr treatment (Figure 3C and Figure S3C), but not for longer time treatments with Acr (3–24 h), indicating that Acr may affect different levels of rRNA generation. This is consistent with our observations that rDNA/rRNA damages induced by Acr lead to the abolition of rRNA transcription, ribosomal assembly and eventually global translation (Figures 4–5 and Figure S4). However, localization of B23 in the Act D-exposed nucleoli was diminished and nucleoli were further increased in compactness and segregation to form nucleolar caps due to inhibition of rRNA synthesis (Figure 3A and 3B).

A widely accepted mechanism of p53 checkpoint activation after alteration of ribosome biogenesis is that it causes leakage of ribosomal proteins including L5, L11, L23 and S7, and that these RPs might bind to MDM2 causing MDM2 dysfunction in mediating p53 degradation [32–36]. In this study, we found that in p53-active A549 cells, inhibition of rRNA transcription by Acr induced p53 stabilization, increased binding of RPL11 with MDM2 and induced E2F-1 degradation (Figure 8A); these results are consistent with the current understanding that in p53 proficient cells altered ribosome biogenesis inhibits cell proliferation through the activation of the RP-MDM2-p53-E2F-1 pathway. We found Acr treatment causes reduction of E2F1 in p53-active A549 cells and p53-inactive HeLa cells. In p53-inactive cells, MDM2 does not bind with E2F-1 but binds with RP11 after Acr treatment. The degradation of the E2F-1 protein has previously been shown to be hindered by its interaction with MDM2, which acts by inhibiting E2F-1 ubiquitylation and subsequent proteasomal degradation [31]. Our results show that the downregulation of E2F-1 protein in Acr-treated cells could be prevented by blocking 26S proteasome, indicating that MDM2 binding protects E2F-1 from degradation (Figure 7). We found that Acr treatment does not affect mRNA levels of E2F-1 supporting the notion that Acr-induced reduction of E2F-1 levels is mediated by translational or a posttranslational control (Figure 7).

The inhibition of rRNA synthesis is not always associated with downregulation of E2F-1. For example, Act D, cisplatin and etoposide – all drugs that inhibit rRNA transcription – cause accumulation of E2F-1 protein by phosphorylation in response to DNA damaging agents [45, 46]. It is worth noting that these drugs are DNA-damaging agents which could activate the checkpoint kinase 2 to phosphorylate E2F-1, resulting in the increases of both its half-life and transcriptional activity [46]. In contrast, it has been demonstrated that silencing TIF1A without using DNA damaging agents, not only inhibits rRNA transcription, but also causes a downregulation of E2F-1 protein [47]. We show that Acr-induced ribosomal stress causes a reduction of E2F-1 and apoptosis in cells with active or inactive p53 (Figure 1 and Figure 7). While it is well established that reduction of E2F-1 can prevent cell proliferation [38], these results support the finding that Acr-induced reduction of E2F-1 inhibits Bcl-2 expression resulting in apoptosis in p53-inactive HeLa cells or in p53-knockdown A549 cells (Figure 8B and 8C and Figure 9C and 9D). However, the extent of apoptosis is much lower in p53-knockdown cells than in control cells. This result indicates p53 plays a crucial role in Acr-induced apoptosis in p53-active cells.

Acr has been shown to trigger either apoptotic or necrotic pathways depending on the cell types, culture conditions, or even medium composition used [1]. For example, Acr has been demonstrated to induce apoptosis in Chinese hamster ovary (CHO) cells, either through the intrinsic pathway which involves cytochrome C release [48] or through the extrinsic pathway by activating death receptors [49]. On the other hand, Acr has also been reported to cause necrotic death in murine FL5.12 proB lymphocytes cells cultured in serum depleted medium [7].

**Figure 10:**
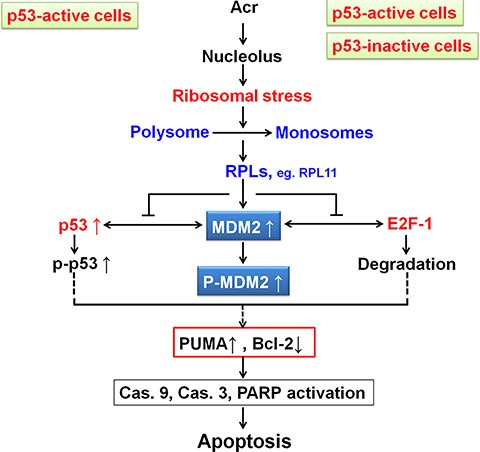
Model of Acr-induced ribosomal stress responses in p53-active and -inactive cancer cells

In summary, we demonstrate here that Acr-induced rDNA/rRNA damages hindered rRNA transcription and processing, which results in MDM2-RPL11 binding, E2F-1 degradation and cellular apoptosis in p53-active A549 cells and p53-inactive HeLa cells. We propose that in p53-active cells activated p53 triggers apoptosis while in p53-inactive cells E2F1 degradation results in suppression of Bcl-2 consequently enhancing Acr-DNA damage induced apoptosis (Figure 10). Our results not only enhance our understanding of the molecular mechanisms of Acr mediated antitumor activity but also may enable the development of better therapeutic strategies for killing cancer cells regardless of their p53 status.

## MATERIALS AND METHODS

### Cell culture and acrolein treatment

Human cervical carcinoma cells (HeLa) and human lung adenocarcinoma cells (A549) (American Type Culture Collection, Manassas, VA) were grown in Dulbecco's Modified Eagle Medium (DMEM) supplemented with 10% FBS and in *RPMI* 1640 Medium supplemented with 10% FBS, respectively. Acrolein (Acr) stock solution (Sigma-Aldrich) was prepared freshly before use. Cells at 70% confluency were washed with PBS buffer (137 mM NaCl, 2.7 mM KCl, 8 mM Na_2_HPO_4_, 1.46 mM KH_2_PO_4_, pH 7.0) and treated with different concentrations of Acr (0–100 μM) in complete culture medium for different times as indicated at 37°C in the dark.

### Knockdown of p53 by RNA interference

RNA interference in A549 was carried out according to the manufacturer's protocol using GenMute siRNA Transfection Reagent (SignaGen Laboratories). The sequence of the p53 siRNA targets p53 mRNA (NM_000546.5) is GACUCCAGUGGUAAUCUAC and siRNA was synthesized by Sigma (St. Louis, MO, USA). The p53 siRNA and control siRNA were transfected at a final concentration of 30 nM for 24 h following by Acr treatment as described above.

### Acr cytotoxicity assay

The Acr cytotoxicity was determined using a modified 3-(4,5- dimethylthiazol-2-yl)-2,5-diphenyl tetrazolium (MTT; Sigma, St. Louis, MO) assay [50] as described previously. Briefly, for MTT assay, cells (5 × 10^3^/ well) were seeded in 96-well plates overnight, and then treated with Acr (0–250 μM, 24 h). The resulting formazan dissolved with DMSO was measured at 570 nm and results were presented as the percentage of the control values. For Lactate dehydrogenase (LDH) leakage assay, cells (2.5 × 10^4^/well) were seeded in 24-well plates overnight and then treated with Acr (0–250 μM, 24 h). LDH activities were measured using the LDH detection kit (Sigma) as described in the manufacturer's protocol. These experiments were performed in triplicates and were repeated at least three times. The extent of cellular damage was calculated based on the percent LDH activity in the supernatants relative to that in the cell lysates.

### ROS production

The generation of pro-oxidants was measured as described previously [51], with modifications. Briefly, A549 or HeLa cells were treated with Acr (0–250 μM) or hydrogen peroxide (H_2_O_2_, 1 mM) as a positive control for one hour. Then, 2′7′- dihydrodichlorofluorescein diacetate (H2DCFDA, 10 μM, Sigma) was added and incubated for 30 min. Levels of pro-oxidants in 10,000 cells were determined by flow cytometry using the FL-1 detector as described previously [52].

### Detection of Acr-dG and 8-oxoG adducts by immunofluorescence assay

For detection of Acr-dG or 8-oxo-dG adducts, immunofluorescent staining were performed as described previously [2]. The following antibodies were used at the noted dilutions: Acr-dG (1:100), 8-oxo-dG (1:100, Abcam. Ab62623), nucleolin (1:500, Cell signaling, #14574), B23 (1:250, Zymed), UBF (1:100, Santa Cruz, sc-9131) or RNA Pol. I (RPA194, 1:100, Santa Cruz, sc-28714). The appropriate fluorophore-conjugated secondary antibodies (1:200, FITC or Rhodamine; Molecular Probes) were used and immunofluorescent images of the fixed cultures were viewed with a fluorescence laser-scanning confocal microscope (Olympus FV10i, Center Valley, PA). The immunofluorescent image acquisition times for the DAPI, Rhodamine and FITC channels, respectively, were kept constant over all samples. Staining specificity was determined by pre-incubating anti-Acr-dG or anti-8-oxo-dG antibody with a 15 to 20-fold excess of soluble Acr-dG and 8-oxodeoxyguanosine (Berry & Associates, Inc., city and state). To investigate if Acr-dG or 8-oxo-dG adducts were preferentially formed in DNA or RNA, Acr-treated cells on coverslips were pretreated with DNase I (0.1 mg/ ml in PBS for 10 min at RT; Sigma) or RNase (1 mg/ ml in PBS for 10 min at 37°C; Invitrogen) before fixation. After enzyme treatment, the remaining DNA or RNA was evaluated by immunofluorescence assay for Acr-dG and 8-oxo-dG adducts as described above.

### Ribosomal profiling assay

The polysome assay was carried out using cell lysate from HeLa or A549 cells (1 × 10^8^) treated with Acr (0–100 μM, 3 h) as described previously [53]. The lysate was prepared in buffer A containing 25 mM HEPES (pH 7.5), 400 mM KOAc, 5 mM Mg (OAc)_2_, 2% TritonX-100, 0.2 mM cycloheximide, and 40 U/ml RNasin (Invitrogen). A 12-ml 10–50% sucrose density gradient containing buffer (20 mM Tris–HCl, pH7.5; 50 mM KCl; 3 mM MgCl_2_) was used for the analysis. The polysome profile was monitored by absorbance at 254 nm using an ISCO auto fractionation instrument and starting with the free material followed by 40S ribosomal subunit detection and continuing through to polyribosome complexes. Aliquots were taken from each fraction and subjected to the phenol–chloroform extraction to allow rRNA analysis.

### Western blot analysis

HeLa or A549 cells were treated with Acr (0–100 μM, 3 h), cell lysates were prepared and analyzed as described previously [4]. Briefly, blots were probed with a monoclonal antibody against MDM2 (1:1000, Abcam, ab178938), p-MDM2 (Ser166, 1:1000, Cell signaling, #3521), E2F-1 (1:1000, Cell signaling, #3742), p-p53 (Ser15, 1:1000, Cell signaling, #9284), p53 (1:1000, Calbiochem) and RPL11 (3A4A7, Thermo Scientific) at 4°C for overnight following by horseradish peroxidase-conjugated secondary IgG (1:3,000; Millipore) for 1 h at room temperature. The immunoreaction was visualized using Enhanced Chemiluminescence (ECL) (Millipore Corporation, Billerica, MA). The bound primary and secondary antibodies were stripped by incubating the membrane in stripping buffer (100 mM 2-mercaptoethanol, 2% SDS) for 30 min at room temperature. The membrane was then re-probed with β-actin antibody (1:5,000; Millipore [clone C4]).

### Immunofluorescent detection of rRNA synthesis

Immunodetection of nascent rRNA was performed by incorporation of 5-fluorouridine (5-FU), according to the method described [54]. Briefly, cells growing on coverslips were incubated with 2 mM 5-FU (Sigma) for 15 min, then washed with cold PBS and fixed in 4% paraformaldehyde and 1% Triton X-100 in PBS for 10 min. Subsequently, the cells were immunofluorescently stained with a specific monoclonal antibody for halogenated uridine (1:400, Sigma [BU-33]). Mounting and nuclei counterstaining and immunofluorescent image were performed as describe as above. Quantification of incorporation of 5-FU into rRNA was using Olympus cellSens^™^ Dimension software (Olympus Life Science).

### Chromatin immunoprecipitation (CHIP) assay

For ChIP assays, cells (5 × 10^6^) were grown in 15-cm dishes overnight. After treatment of Acr, DNA was cross-linked with 1% formaldehyde at room temperature for 10 min and assays performed using 10^6^ cells per immunoprecipitation as described in Chromatin Immunoprecipitation (ChIP) Assay Kit (Millipore). Cells were lysed in the presence of protease inhibitors and chromatin was fragmented to 200–1000 bp by sonication (high power, 20 cycles of 30 seconds with 30 seconds between pulses). Immunoprecipitations were performed with 2 mg of UBF (1:100, Santa Cruz, sc-9131) or RNA Pol I (RPA194, 1:100, Santa Cruz, sc-28714), or 2 mg of normal rabbit IgG (Santa Cruz, sc-2027). Complexes were collected with protein G agarose (GE, 17-0618-01). De-crosslinked DNA was purified and eluted in 50 ml of elution buffer of which 2 ml was used for PCR. Primers (5′-3′) were CGATGGTGGCGTTTTTGG and CCGACTCGGAGCGAAAGATA for the rRNA promoter region; and CGACGACCCATTCGAACGTCT and CTCTCCGGAATCGAACCCTGA for the rRNA transcribed region. Samples were analyzed in triplicate using the SYBR green dye on the StepOnePlus^™^ Real-Time PCR System (Applied Biosystems). To calculate the percentage of total DNA bound, unprecipitated input samples from each condition were used as reference for all qPCR reactions.

### Immunoprecipitation (IP) assay

For IP studies, cells were washed and scraped in PBS, then suspended in IP lysis buffer (20 mM Tris-HCl (pH7.4), 170 mM NaCl, 13 mM MgCl_2_, 0.5% NP40) and dounced 30 times in a Dounce homogenizer. After centrifugation at 16,100 × g, 4°C for 15 min, the protein concentrations in the supernatant were measured using BCA protein assay kit (Pierce, Rockford, IL). IP procedure was followed according to manufacturer's instructions (Dynabeads^®^ protein G, Invitrogen). Briefly, 2 μg of MDM2 (ab16895, Abcam) or IgG (Santa Cruz Biotechnology Inc) antibody in 300 ml of IP lysis buffer was incubated with 100 μl of dynabeads protein G for 2 h on rotating platform at 4°C. After removing antibody solution using the Dynal magnet system, 1 mg of protein samples in 300 ml of IP lysis buffer were added and incubated overnight on rotating platform at 4°C. Beads were then washed (using the Dynal magnet system) three times with 0.5 ml of ice cold IP buffer. After the last wash, beads were centrifuged and last traces of buffer were removed using a micropipette. Antibodies/protein complexes were eluted with 1× SDS sample buffer followed by western blotting.

### Quantitative evaluation of the 28S and 18S rRNA transcripts

Total RNA of A549 or HeLa cells treated with Acr (0–100 μM, 3 h) was extracted using Trizol (Invitrogen) according to manufacturers' instructions. The 28S and 18S RNA subunits were visualized by loading in a 1% agarose gel stained with ethidium bromide an equal fraction (10%) of the total quantity of the obtained RNA. The intensity of the bands was evaluated with the densitometric software UVP^™^ Doc-It^™^ LS Image Analysis Software.

### Quantitative real-time RT-PCR

Total RNA was isolated from harvested cells using *TRIzol*^®^ *Reagent* (Thermo Fisher Scientific). Reverse transcription was RevertAid Reverse Transcriptase (Thermo Fisher Scientific) according to manufacturers' instructions. Subsequent real-time RT-PCR analysis of cDNA were performed in triplicate using the SYBR green dye on the StepOnePlus^™^ Real-Time PCR System (Applied Biosystems). The primers (5′-3′) were CTCCGTTATGGTAGCGCTGC and GCGGAACC CTCGCTTCTC for 45S; CGACGACCCATTCGAAC GTCT and CTCTCCGGAATCGAACCCTGA for 18S; GCCACTGACTCTGCCACCATAG and CTGCCCATC CGGGACAAC for E2F1; CCGTCTAGAAAAACCTGCC and GCCAAATTCGTTGTCATACC for GAPDH. To calculate the relative RNA expression, GAPDH was used as an internal control for all qRT-PCR reactions and compared with control groups.

### Flow cytometry analysis of cell cycle phases

Cells (5 × 10^5^/ 6-well plate) treated with Acr (75 μM, 0–24 h) as previously described [55]. After harvested cells were washed twice in ice-cold PBS and fixed in ice-cold 70% ethanol for 30 min or overnight at 4°C. Cells were washed in PBS and digested with DNase-free RNase A (50 U/ ml) at 37°C for 30 min. Before flow cytometry analysis, cells were resuspended in 500 μl propidium iodide (PI, 10 μg/ ml; Sigma) for DNA staining. PI staining was used to measure for cell cycle status using a Becton-Dickinson FACScan instrument and Cell Quest software.

### Determination of phosphatidylserine (PS) externalization by Annexin V-FITC staining

Cells (1 × 10^6^/ 10-cm dish) treated with Acr (0–100 μM, 24 h) were analyzed by FITC Annexin V Apoptosis Detection Kit I (BD Biosciences Canada, Mississauga, ON, Canada) according to manufacturers' instructions. Briefly, Acr-treated cells were washed with PBS and resuspended in 1 ml of binding buffer (10 mM HEPES/ NaOH, pH 7.5, 140 mM NaCl, and 2.5 mM CaCl_2_). A volume of 500 μl of cell suspension was incubated with 5 μl of Annexin V-FITC and 10 μl of propidium iodide (PI) for 10 min at room temperature in the dark. Cells (10,000) were then analyzed by flow cytometry. Annexin V-FITC fluorescence was detected on the FL-1 detector and PI fluorescence on the FL-2 detector. Four populations of cells were analyzed: live control cells (Annexin V−/PI−); early stage apoptotic cells (Annexin V+/PI−); late stage apoptotic cells (Annexin V+/PI+); necrotic cells (Annexin V−/PI+). The results are reported as the fraction of total apoptotic cells (early and late stage apoptosis) and necrotic cells.

### Protein synthesis evaluation

The global protein synthesis was analyzed using Cayman's protein synthesis assay Kit (Cayman chemical, # 601100), following the manufacturer's protocol. Briefly, the protein synthesis rate was evaluated by incorporation of puromycin analog o-propargyl-puromycin (OPP) added to the Acr-treated HeLa or A549 cells resuspended in complete medium. Upon application to cells, the OPP probe incorporates into the C-terminus of translating polypeptide chains, thereby stopping translation. These truncated C-terminal alkyne-labeled proteins are then subsequently detected via copper-catalyzed click chemistry using 5 FAM-Azide following by flow cytometry. Histograms show the values (mean ± s.d.) of three independent experiments.

### Statistical analyses

Student's *t*-tests were used to determine statistical significance, and two-tailed *P*-values are shown. A minimum of three independent replicate experiments was performed to justify the use of statistical tests.

## SUPPLEMENTARY MATERIALS


