# Supplementary material for: Acrolein preferentially damages nucleolus eliciting ribosomal stress and apoptosis in human cancer cells
Source: Oncotarget. 2016 Oct 12;7(49):80450–64. doi: 10.18632/oncotarget.12608 (PMC5348333; doi:10.18632/oncotarget.12608)
Supplement: Supplementary file 1 [file oncotarget-07-80450-s001.pdf]

Acrolein preferentially damages nucleolus eliciting ribosomal stress and apoptosis in human cancer cells

Supplementary Materials

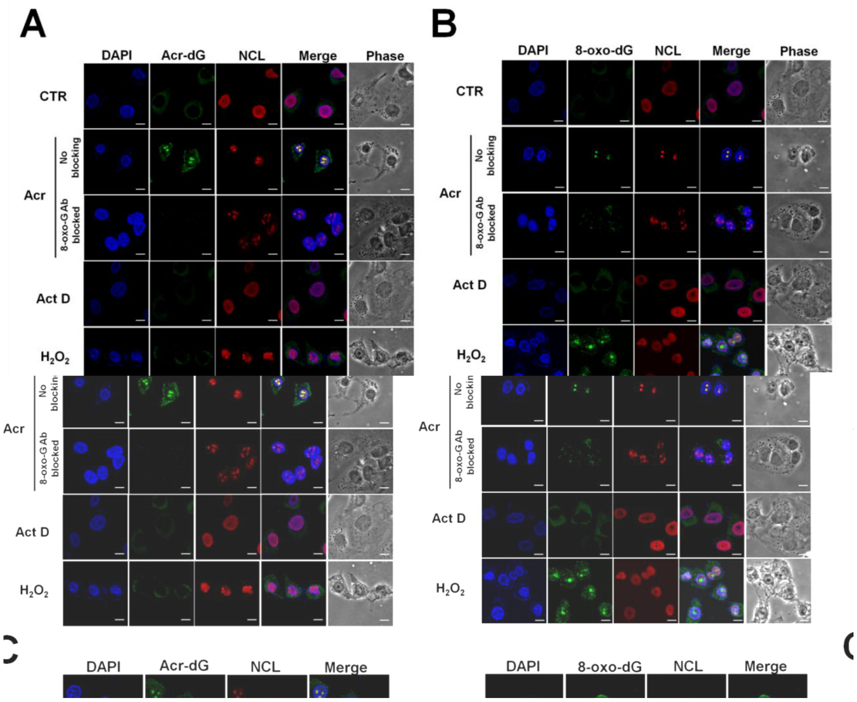

Supplementary Figure S1: Acrolein induces Acr-dG and 8-oxo-dG adducts in A549 cells. The methods for immunofluorescence staining of Acr-dG and 8-oxo-dG adducts in Acr-treated A549 cells were the same as in Figure 2. Cells were treated with Acr (75 µM for 3 h), fixed, stained with (**A**) anti-Acr-dG and (**B**) 8-oxo-dG antibody followed by goat anti-mouse Alexa 488 -conjugated secondary antibody and then examined by microscopy. Nucleolin (NCL) was used to stain nucleoli (A and B). (**C**) Effect of DNase and RNase treatment on Acr-dG and 8-oxo-dG adduct detection in Acr-treated A549 cells. Acr-treated cells on glass slide were treated with DNase (0.1 µg/ µl, RT for 10 min) or RNase (1 µg/ µl, 37°C for 10 min) and fixed following by immunofluorescence staining of Acr-dG and 8-oxo-dG adducts.

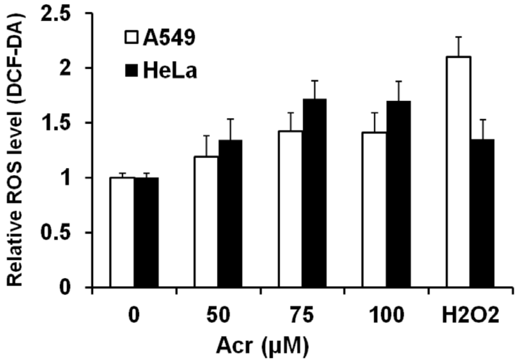

Supplementary Figure S2: Acrolein increases intracellular ROS production in A549 and HeLa cells. DCF assay for intracellular ROS production in A549 and HeLa treated with different concentrations of Acr (0–100 µM) and H_2O_2 (1 mM) for 1 h was described in Materials and Methods.

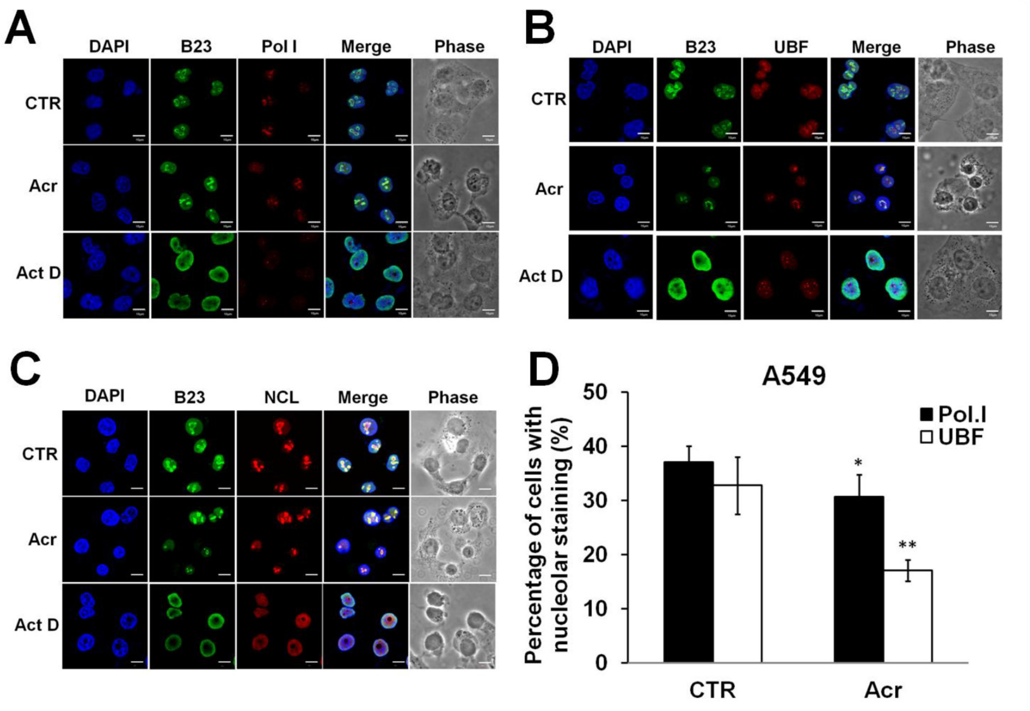

Supplementary Figure S3: Acrolein decreases nucleolar RNA Pol I and UBF translocation and induces nucleolar disintegration in A549 cells. (**A** and **B**) Immunofluorescence staining of RNA Pol I and UBF in Acr- treated A549 cells. Cells were treated with Acr (75 μM for 3 h) or Act D (20 ng/mL for 3 h), fixed, stained with RNA Pol I and UBF antibody followed by goat anti-rabbit Rhodamine-conjugated secondary antibody and then examined by microscopy. B23 was used to stain nucleoli. (**C**) Effect of Acr (75 μM) or Act D (20 ng/ml) treatment on nucleolar structure. A549 cells were treated with Acr (75 μM) or Act D (20 ng/ml) for 3 h, stained with B23 and NCL antibody then immunofluorescen labeled second antibody the same as in Figure 3. Nuclei were counter-stained with DAPI. Scale bar: 10 μm. Quantification of nucleolar Pol I or UBF was showed in (**D**). Histograms show the values (mean ± s.d.) of three independent experiments. **P* value < 0.05, ***P* value < 0.01. Student's *t*-test was used to calculate significance between control and treatment.

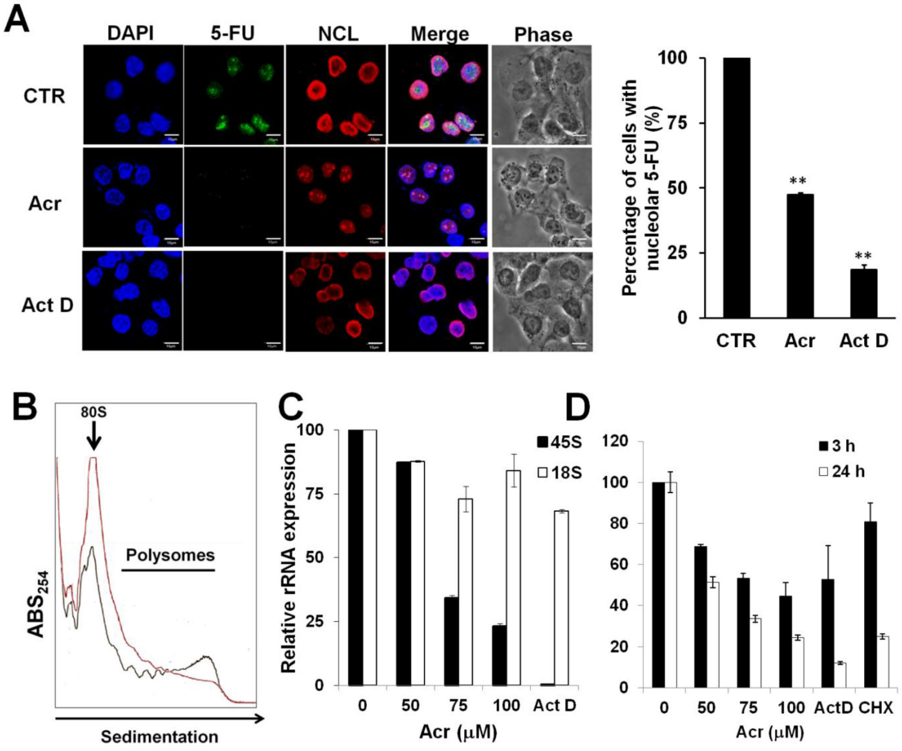

Supplementary Figure S4: Acrolein interrupts rRNA synthesis, ribosome assembly and global protein synthesis in A549 cells. (**A**) Effect of Acr or Act D treatment on rRNA synthesis in A549 cells. Cell were treated with Acr (75 μM) or Act D (20 ng/ml) for 3 h and labeled with 5-fluorouridine (5-FU) for 15 min. The incorporation of 5-FU was revealed by specific FITC-conjugated monoclonal antibodies. DAPI counter-stained nuclei. Scale bar: 10 μm. Quantification of nucleolar 5-FU is showed in the right panel. Histograms show the values (mean ± s.d.) of three independent experiments. Symbols: * and ** represent *P* value < 0.05, and < 0.01. (**B**) Polysome assays derived from A549 cells treated with Acr (75 μM, 3 h). Regions represent the 80S monosomes and polysomes (underlined by a bar) were shown. (**C**) Effect of Acr treatment on the expression of 45S and 18S rRNA expression in A549 cells. Cells were treated with Acr (0–100 μM, 3 h) and the synthesis of 45 S and 18 S using real-time RT-PCR analysis. Histograms show the values (mean ± s.d.) of three independent experiments. Scale bar: 10 μm. (**D**) Total protein synthesis evaluated by 30 minutes, puromycin analog O-Propargyl-puromycin (OPP) incorporation into A549 cells treated with Acr (0–100 μM), Act. D (20 ng/ml) or cyclohexymide (CHX) (50 μM) for 3 or 24 h. Protein synthesis assay was described in materials and methods. Histograms show the values (mean ± s.d.) of three independent experiments. **P* value < 0.05, ***P* value < 0.01. Student's *t*-test was used to calculate significance between control and treatment.
